# Presentation and surgical management of a gossypiboma presenting with small bowel obstruction

**DOI:** 10.1007/s12328-021-01400-y

**Published:** 2021-03-31

**Authors:** George Ryan, Michal Kawka, Janakan Gnananandan, Vincent Yip

**Affiliations:** 1 Department of Colorectal Surgery, Royal London Hospital, London, UK; 2 Department of Medicine, Imperial College London, London, SW7 2AZ UK; 3 Department of Hepato-Pancreato-Biliary Surgery, Royal London Hospital, London, UK

**Keywords:** Gossypiboma, Transmural migration, Intestinal obstruction, Case report, Emergency surgery

## Abstract

Gossypiboma is a cotton-based foreign body retained within the human body following a surgical procedure. Transmural migration of intra-abdominal gossypiboma into the small bowel is rare; however, it can present with life-threatening complications. We report a case of a 28-year-old male who presented with small bowel obstruction due to gossypiboma, 11 years after the initial surgical procedure. Due to the size of the retained surgical swab, 40 cm × 40 cm, an open surgical approach was preferred. Following removal of the retained swab and bowel reconstruction, the patient was followed in clinic and discharged without complications. Staff education and adherence to operating room record-keeping protocols can prevent gossypiboma. To the best of our knowledge such a long interval between the initial surgery and presentation of gossypiboma that large has not been previously reported in the literature.

## Introduction

Gossypiboma, derived from the Latin word ‘gossypium’ meaning cotton and Swahili word ‘boma’ meaning place of concealment, describes a retained non-absorbable surgical material composed of a cotton matrix [1]. The incidence of gossypiboma is unclear due to the medico-legal ramifications of reporting. Its presenting symptoms vary from mild abdominal pain to significant complications, including bowel perforation, bowel obstruction, peritonitis, sepsis or fistula formation [2]. Transmural migration of an intra-abdominal gossypiboma is rare; migration into the stomach, ileum, small bowel, colon, bladder, vagina, pericardium, nose, urethra and diaphragm was previously reported [3, 4]. To our knowledge, this is the 6th reported case of transmural migration of a gossypiboma into the small bowel; however, previous cases describe retrieval of small swabs by endoscopic methods. This case describes the retrieval of a 40 cm × 40 cm surgical swab by an open surgical approach in a 28-year-old male, 11 years after the initial surgery.

## Case

A 28-year-old man presented to the Accident and Emergency department of a University Teaching Hospital in London with a 1-month history of left upper quadrant pain, worsening over the last week. He had been “retching” and “spitting” the day before, with reduced appetite during the preceding days. He reported no history of chest pain, breathlessness, cough or fever. The patient had opened his bowels the day before. The patient did not have any past medical history but had two laparotomies, both when he was younger, for reasons he was unable to recall in detail. The first laparotomy, when the patient was 8 months old, was performed possibly for pyloric stenosis, while the second at 14 years, of age possibly for bowel obstruction. He did not take regular medication and had been living and working independently prior to admission.

**Fig. 1:**
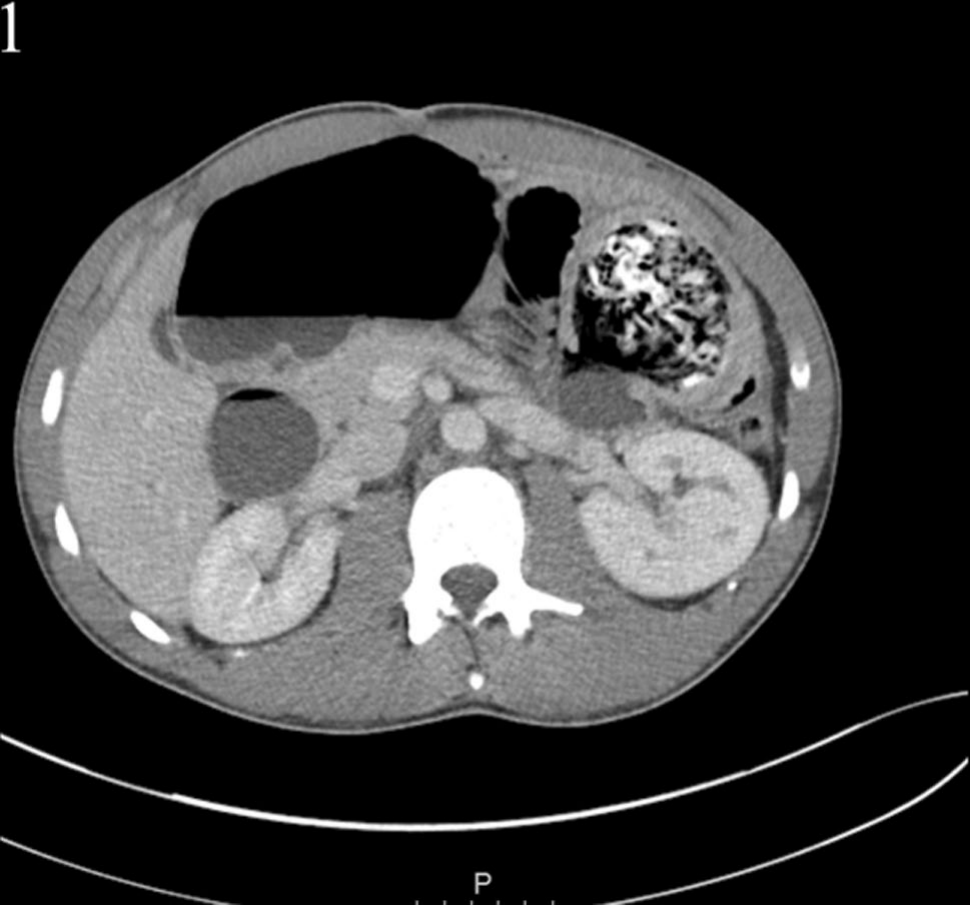
Axial CT image showing proximal duodenal and gastric distension secondary to heterogenous intraluminal mass at the duodenal jejunal flexure/proximal jejunum which is suspicious for gossypiboma

**Fig. 2:**
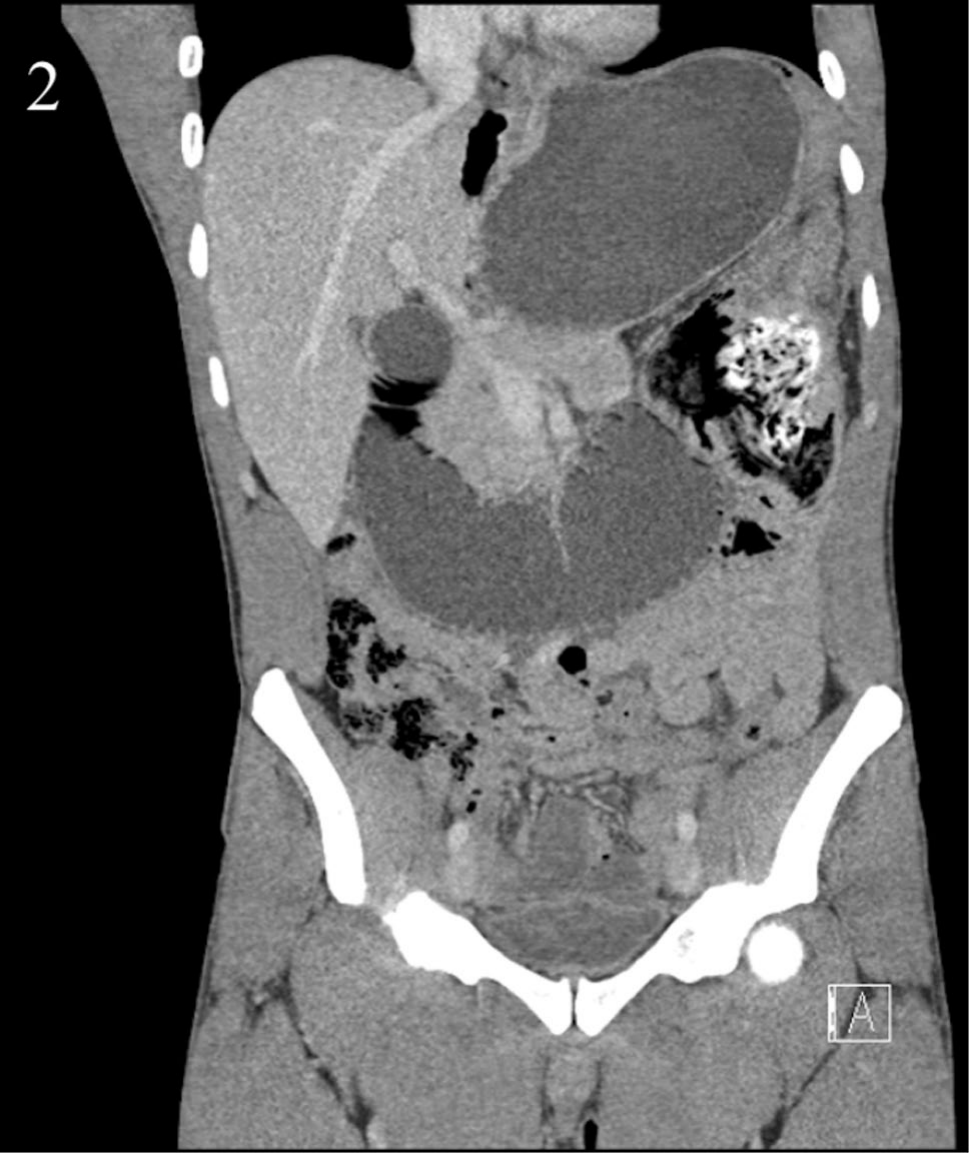
Coronal CT image showing proximal duodenal and gastric distension secondary to heterogenous intraluminal mass at the duodenal jejunal flexure/proximal jejunum which is suspicious for gossypiboma

Patient's observations were stable; heart rate 75 bpm, respiratory rate 20 br/min, oxygen saturations 98%, on room air, temperature 36.5 °C, blood pressure 136/80 mmHg. On examination, the abdomen was soft and tender along the left flank and left upper quadrant but not distended, with no signs of peritonism or hernia. Laboratory investigations were unremarkable other than a white blood cell count of 12.3 × 10^3^/mL and C-reactive protein of 75 mg/L. The urine dip was negative for leukocytes and blood. The abdominal radiograph revealed a ‘double bubble’ sign in the left upper quadrant. Computed tomography of the abdomen and pelvis was performed, and the radiologist reported “proximal duodenal and gastric distension secondary to heterogenous intraluminal mass at the duodenal jejunal flexure/proximal jejunum which is suspicious for gossypiboma” (Figs. 1, 2).

**Fig. 3:**
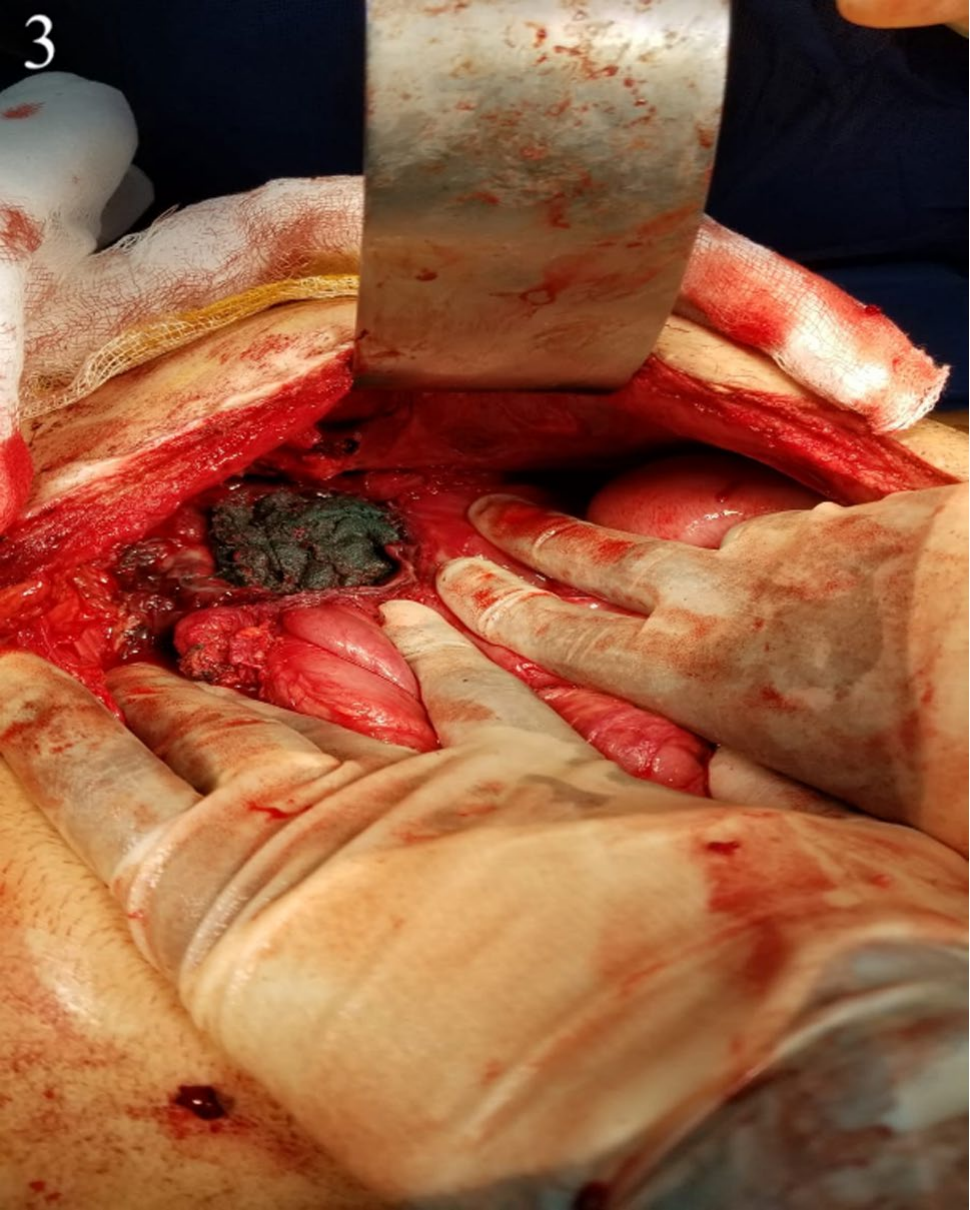
Intra-operative photograph showing gossypiboma in situ and perforation through the jejunum

**Fig. 4:**
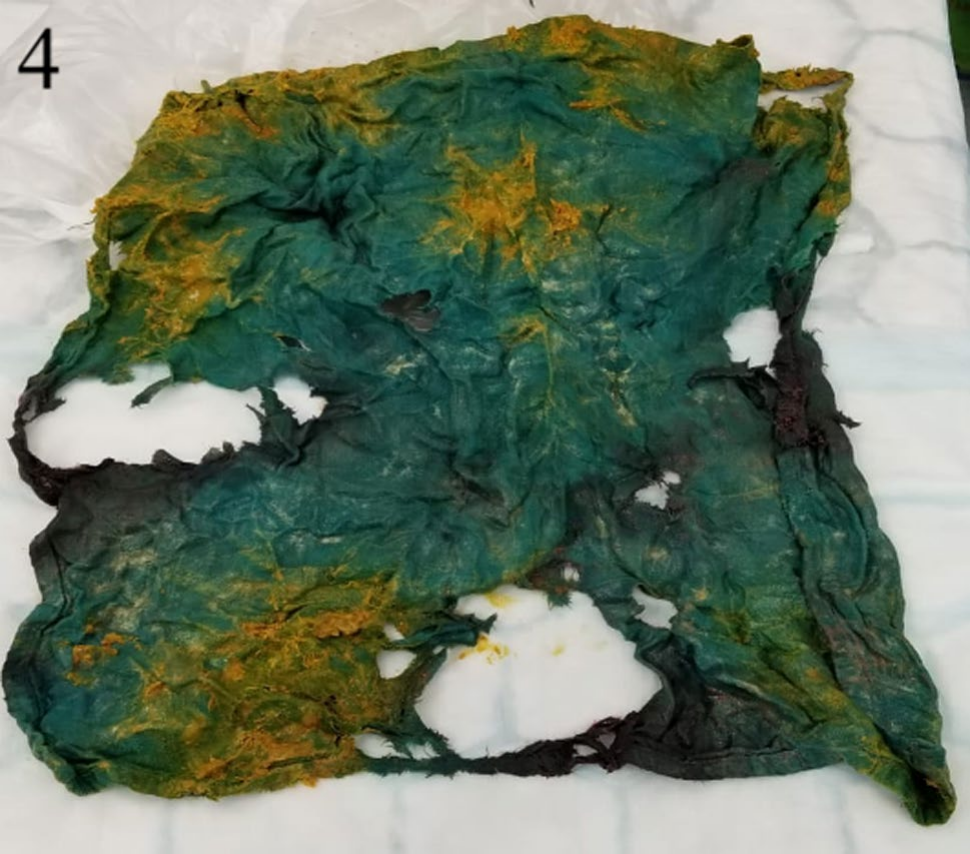
Gossypiboma removed from abdomen, 40 × 40 cm

The patient underwent a laparotomy which revealed significant adhesions and a 15 cm × 10 cm mass within the jejunum, 10 cm distal to the duodenal jejunal flexure. This was adherent to the splenic flexure, descending colon and two separate loops of small bowel. Adhesiolysis was performed, and a 10 cm segment of adherent jejunum and splenic flexure were divided and removed en-bloc. A side-to-side stapled jejuno-jejunostomy and a side-to-side stapled colo-colonic anastomosis were fashioned. A 40 cm × 40 cm surgical green huck towel was retrieved from the abdomen (Figs. 3, 4).

The patient was transferred to the Acute Critical Care Unit, where three days later, he deteriorated clinically. He was returned to theatre due to concern of an anastomotic leak. A laparotomy was performed revealing a small serosal tear in the bowel, which was oversewn. Both anastomoses were intact with adequate lumens. The patient made an unremarkable recovery and was discharged eleven days later. Six weeks after discharge, he was seen in the clinic where no further concerns were identified, and the patient was discharged from follow-up care.

## Discussion

Gossypiboma is a rare occurrence with a reported incidence of 1:1000–1:10,000 in intra-abdominal operations. Transmural migration occurs in a small proportion of these. This case describes transmural migration of gossypiboma, which presented 11 years after surgery, to our knowledge, the literature describes no case of similar longevity.

Gossypiboma migrates in four stages: foreign-body reaction, secondary infection, mass formation and remodelling [4]. Two different types of pathological foreign body reactions can occur: a fibrinous response creating adhesions and encapsulation or an exudative process leading to abscess formation [4]. The most commonly affected site is the small intestine due to its relatively large surface area and thin walls which offer little resistance to invasion.

The interval between the operation and presentation of gossypiboma depends on the anatomical location and pathological process. Approximately one-third of gossypiboma patients remain asymptomatic, with the foreign body detected incidentally. Most commonly, as was in our case, the fibrinous response encapsulates the gossypiboma in omentum and adhesions resulting in non-specific symptoms such as abdominal pain and nausea. However, if the systemic inflammatory response is invoked, more significant symptoms such as nausea, vomiting and diarrhoea can appear. If the gossypiboma is in close proximity to the bowel, the effects of local inflammation combined with pressure can lead to erosion through the wall resulting in leakage of bowel contents potentially causing obstruction of the lumen. The internal is also influenced by the age of the patient, co-existing medical conditions and healthcare system in which the condition is managed. Asymptomatic gossypiboma can be also incidentally detected on routine medical imaging or during investigations of co-existing medical conditions [5]. These occurrences are more common to occur in older patients and in healthcare systems with wider access to imaging technologies [5]. As such, the young age of the patient, lack of co-morbidities and no imaging studies contribute to explaining the 11-year interval between initial surgery and the presentation in our case.

Gossypiboma should be removed urgently to avoid further complications and legal ramifications. Open surgery is a common approach; however, depending on location, size of foreign body and skill of the operator, minimally invasive techniques such as endoscopy or laparoscopy have proven effective [1]. In cases where transmural migration is suspected, endoscopic treatments are mostly unsuccessful [6].

Prevention is another important part of disease management. Patients undergoing emergency surgery, those with high body mass index, long operations, inexperienced staff or unexpected change in the procedure are at risk for retained surgical materials [1]. Simple precautions such as staff education, performing the WHO (World Health Organisation) [7] checklist, tagging the sponges with markers or multiple perioperative counts of sponges and materials reduce the incidence of gossypiboma. If there are concerns over a retained surgical swab, the use of intra- or post-operative radiography to assist in confirming the suspicion is indicated [8]. This is enabled by the aforementioned ‘tagging’ of modern surgical swabs and sponges, which will appear as hyperdense, linear structures on plain radiographs, if retained [8].

## Conclusion

This report has described the rare case of a gossypiboma displaying transmural migration presenting 11 years after surgery. It depicts how a significantly sized foreign body can be retained and remain dormant for a long time and still present with life-threatening complications requiring urgent operative intervention.
